# Construction of a Hydrogel Pectin-Based Triglyceride Optical Biosensor with Immobilized Lipase Enzymes

**DOI:** 10.3390/bios9040135

**Published:** 2019-11-13

**Authors:** Uswatun Hasanah, Nor Diyana Md Sani, Lee Yook Heng, Rinaldi Idroes, Eka Safitri

**Affiliations:** 1 Graduate School of Mathematics and Applied Sciences, Universitas Syiah Kuala, Banda Aceh 23111, Indonesia; uswatun.hasanah@utu.ac.id; 2 Department of Fisheries, Faculty of Fisheries and Marine Sciences, Universitas Teuku Umar, West Aceh 23615, Indonesia; 3 Sanichem Resources Sdn. Bhd. No 7 & 7A Jalan Timur 6/1A Mercato @Enstek, Bandar Enstek NSN 71060, Malaysia; diyanasani@yahoo.com; 4 School of Chemical Sciences and Food Technology, Faculty of Science and Technology, Universiti Kebangsaan Malaysia, Bangi SGR 43600 UKM, Malaysia; leeyookheng@yahoo.co.uk; 5 Department of Chemistry, Faculty of Mathematics and Natural Sciences, Universitas Syiah Kuala, Banda Aceh 23111, Indonesia; rinaldi.idroes@unsyiah.ac.id; 6 Department of Pharmacy, Faculty of Mathematics and Natural Sciences, Universitas Syiah Kuala, Banda Aceh 23111, Indonesia

**Keywords:** triglyceride optical biosensor, pectin, chromoionophore

## Abstract

A novel and simple optical biosensor to detect triglycerides (TGs) has been successfully constructed by using pectin hydrogel membrane as the indicator pH and chromoionophore ETH 5294 (CI), with lipase as the catalyst. The enzymatic working system against TGs releasing H^+^ ions will affect the color absorbance of CI. The characterization results show that a TG biosensor has the optimum condition and sensitivity at the phosphate buffer concentration of 50 mM, pH 7, and enzyme loading of 60 μg. The biosensor works at the tripalmitin (TP) concentration range of 100–400 mg/dL. With the sensitivity of 0.001 (∆A/(mg/dL)), the biosensor response reaches stability after five minutes, and the limit of detection (LOD) of the TG optical biosensor is 15 mg/dL. Relative standard deviation (RSD) in a reproducibility test was 2.5%, with a 15-day lifespan.

## 1. Introduction

A triglyceride (TG), also known as a type of biolipid, is a triester of glycerol bound to three fatty acids, with varying saturation degrees between 0 and 6 [1]. TGs play an important role in cellular metabolism as an energy source for dietary lipid transportation [2]. In the human body, TGs are a health indicator, where their normal serum concentration range is from 40 to 150 mg/dL [3]. Elevated TG concentrations in the body may result in cardiovascular and coronary heart diseases [4,5,6,7]. Therefore, due to this health concern, more effective ways to determine TG concentrations in food, beverage, and cosmetic products must be studied.

At present, with the rapid advancement of science and technology, cutting-edge equipment has been developed for the detection of TGs. These include colorimetry [8,9], spectrophotometry [10,11,12,13,14], chromatography [15,16], fluorometry [17], titrimetry [18], nuclear magnetic resonance [19], and enzymatic colorimetry [20]. Nevertheless, most of this mainstream equipment operates in a time-consuming and expensive fashion, not to mention requiring highly skilled technicians. Therefore, a simple and selective avant-garde method for TG analysis is in high demand. Biosensors have received considerable attention from researchers for qualitative TG analysis of late. Scientists are particularly interested in looking into employing electrochemical analysis techniques [21,22,23], using multienzyme working systems [24,25] to develop these biosensors.

Previous electrochemical TG biosensor design has included TG measurement based on pH change from the hydrolysis of triglycerides by enzymes [26,27]. However, the change of pH in a potentiometric system will be highly affected by the presence of interference ions in a sample. Hence, we have chosen to construct an optical biosensor instead, so that this flaw will be eliminated, and our biosensor will be unaffected by ionic interferences.

In the construction of a biosensor, the immobilizing matrix plays an important role in maintaining biosensor stability during an analyte reaction and imparting a longer shelf life. A biosensor matrix can be constructed from synthetic and inorganic polymers, such as silicon [28,29,30] and polyvinyl chloride [31]. However, membranes from synthetic materials possess inefficient permeability, and in the long run are detrimental to the environment. Hence, biopolymeric materials like pectin [32] will be an excellent choice for a biosensor matrix. Biopolymers are superior in terms of biocompatibility, capability of enzyme absorption, thermal stability, and not to mention environmentally friendliness. Pectin, for instance, has been recognized for its hydrogel properties, which can facilitate the interaction of the active compounds and analytes within the matrix [32,33].

In this research, we report the development of a triglyceride, optical, bio-enzymatic biosensor, using pectin hydrogel membrane as its enzyme immobilizing matrix and containing chromoionophore ETH 5294 (CI) as a pH indicator. The hydrogel membrane is prepared with pectin, crosslinked with CaCl_2_ and CI as the first layer. The second layer consists of lipase enzymes immobilized via entrapment, which acts as the specific biocatalyst for TG hydrolysis. The products of TG hydrolysis by lipase are glycerol and fatty acids.

**Figure 1:**
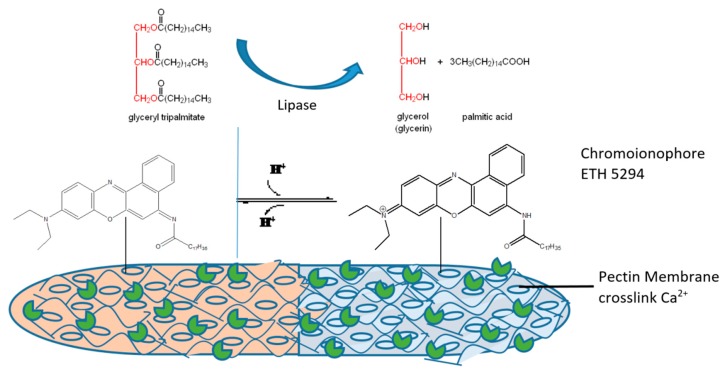
Triglyceride optical biosensor, developed from immobilized lipase enzymes via entrapment and adsorption on the pectin hydrogel membrane surface.

The enzymatic reaction is known to produce H^+^ ions from fatty acids, leading to a pH change that causes the CI to be protonated. CI changes color from pink (deprotonated form) to blue (protonated form) on the pectin hydrogel membrane surface, and can be observed with a UV-VIS spectrophotometer. To the best of our knowledge, there is no research reporting triglyceride determination using a biosensor based on optical calculations. The design of the TG optical biosensor is illustrated in Figure 1.

## 2. Materials and Methods

### 2.1. Materials and Instruments

Chemicals used in this research include CI, monopotassium dihydrogen phosphate (KH_2_PO_4_), dipotassium phosphate (K_2_HPO_4_) from Fluka (Steinhem, Germany) and *Candida antartica* lipase b enzyme from *Aspergillus oryzae* (~9 U/Mg), as well as Glycerol tripalmitin (TP) ≥ 85%, pectin, absolute ethanol (C_2_H_5_OH), and calcium chloride (CaCl_2_) from Sigma Aldrich (St Louis, MO, USA).

The absorbance signal from the biosensor was determined by a Shimazu 1800 UV-VIS spectrophotometer (Kyoto, Japan) and pH buffer controlled with a Thermo Orion Star A2115 pH meter (Waltham, MA, USA). The pectin membrane surface morphology was analyzed with a Zeiss Merlin/Merlin/Gemini’2 Compact/Supra 55VP (Carl Zeiss, Jena, Germany) field emission scanning electron microscopy (FESEM).

### 2.2. Reagent and Solution

The CI reagent was prepared by dissolving 0.4 mg CI in 1 mL ethanol and mixing for 10 min until homogenous. Potassium phosphate buffer solution (PBS) was prepared by mixing monopotassium dihydrogen phosphate (KH_2_PO_4_) with dipotassium hydrogen phosphate (K_2_HPO_4_). The TP stock solution was prepared by diluting TP stock solution with PBS 10 mM at pH 7. Enzyme lipase solution is prepared by dissolving 2 mg lipase in 1000 µL PBS 10 mM (pH 7), stored in a freezer at 4 °C. The 2% hydrogel membrane solution is prepared by mixing 2 grams of pectin into 100 mL CaCl_2_ solution 0.1 M. CaCl_2_ 0.1 M solution is prepared by dissolving CaCl_2_ into PBS 0.01 M solution at pH 7 and heating at 60 °C for 30 min while stirring until homogenous.

### 2.3. Preparation of Triglyceride Optical Biosensor and Biosensor Response Optimization

Our TG optical biosensor was prepared as illustrated by Hasanah et al. [33], with modifications. The TG optical biosensor had two layers: the bottom layer was the pH optical sensor, consisting of CI contained in a pectin membrane (Pectin/CI); meanwhile, the upper layer consisted of lipase enzyme solution. The bottom layer was obtained by dissolving 1 mL pectin 2% and the addition of 400 μL CI solution, which was mixed for 10 min until homogenous. A total of 55 µL of this pectin membrane/CI solution was added onto the surface of a plastic sheet, creating a circle 8 mm in diameter, and was left to dry overnight at room temperature (25 °C). Next, 30 μL lipase enzyme solution was coated onto the dried pectin membrane/CI layer and stored in refrigerator for 24 h at 4 °C. Before use, the pH optical sensor was first converted into its deprotonated form, using TP and PBS 1 M buffer at pH 9. All parameters were conducted using three replicate biosensors. In addition, this biosensor was designed as a disposable, and will not be reused. The biosensor also does not require immersion in a sample, and it only involves dropping small amount of sample (40 μL) onto the surface of the biosensor.

The effect of PBS buffer concentration on the TG biosensor is determined by varying PBS concentrations of 10, 50, and 100 mM at pH 7 and TP concentration from 100–500 mg/dL, measured at a wavelength of 615 nm.

TG biosensor response optimization against the pH change was determined using PBS 50 mM and a TP concentration of 100–500 mg/dL, at a range of pH 6–8.

In order to evaluate the effect of lipase enzyme concentration against the TG biosensor, the lipase concentration was varied between 20–100 µg at a TP concentration of 400 mg/dL in PBS buffer 50 mM, at pH 7.

The response time of the TG biosensor was determined by measuring the biosensor response every minute for 10 min at 400 mg/dL TP concentration

### 2.4. Triglyceride Optical Biosensor Performance Characterization

The performance of the TG optical biosensor probe was evaluated using a few parameters, including dynamic linear range, the limit of detection (LOD), storage stability of the TG biosensor, and its reproducibility.

The dynamic linear range of the TG optical biosensor was constructed against increasing TP as the triglyceride, with concentrations in the range of 100 mg/dL to 500 mg/dL in 50 mM PBS at pH 7. This was followed by the LOD, where three blank absorbance values were taken and calculated based on the equation.

LOD = 3*SD*/*S*

where *SD* is the standard deviation of the blank values and *S* is the slope of the plotted concentration of TP from 100–400 mg/dL versus absorbance using an optimized biosensor.

Next, in order to evaluate the storage stability of this TG biosensor, 50 TG biosensors are produced and stored in a refrigerator at 4 °C. The biosensors were measured periodically, where during each measurement, three biosensors were used. These biosensors were tested with a TP concentration of 400 mg/dL in 50 mM PBS pH 7 for 25 days.

The reproducibility of the TG biosensor is evaluated by measuring 10 TG biosensors and calculating their relative standard deviation (RSD) and sensitivity values.

## 3. Results and Discussion

### 3.1. Characterization of Pectin Hydrogel Membrane

**Figure 2:**
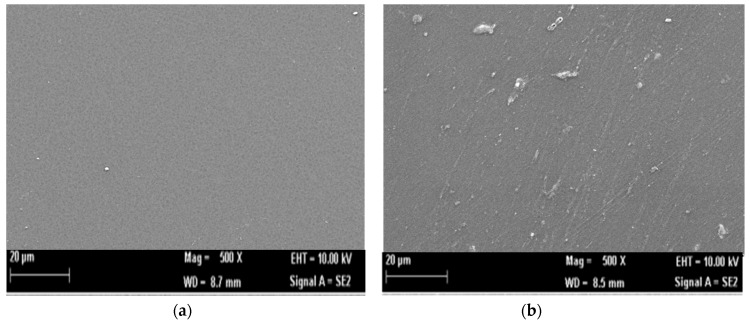

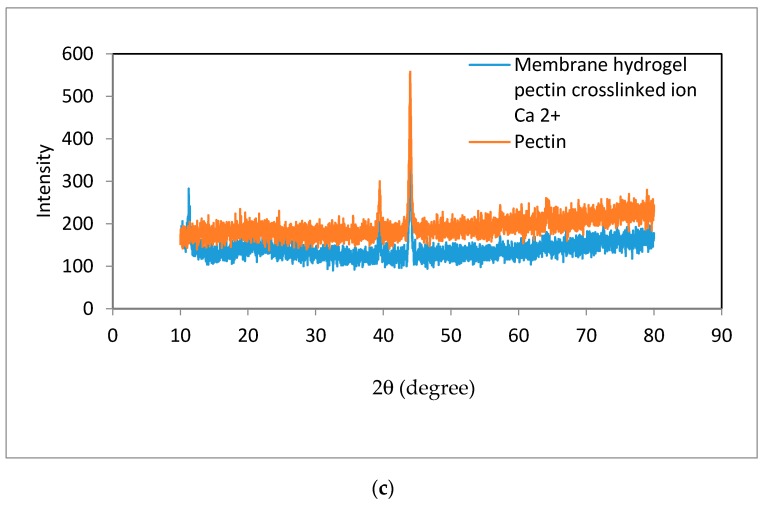
Characterization of pectin hydrogel membrane: (**a**) SEM morphology of pectin hydrogel membrane (pectin + CaCl_2_ + CI); (**b**) SEM morphology of a triglyceride (TG) optical biosensor membrane (pectin + CaCl_2_ + CI + lipase); (**c**) Crystallinity diffractogram of pure pectin and pectin hydrogel membrane.

Pectin hydrogel membrane is made with a CaCl_2_ crosslinker. Ca^2+^ ions can induce pectin to be crosslinked when CaCl_2_ is inserted into pectin solution, thus forming a gel. The obtained hydrogel membrane is colorless, and morphological observation with scanning electron microscopy (SEM) shows that the pectin membrane is homogenous, giving it a smooth surface, which is important for the absorbance value consistency (Figure 2a). On the other hand, CI is added into the pectin hydrogel membrane as an indicator, with lipase enzyme acting as the catalyst for TG biosensor. As seen in Figure 2b, the morphology of a pectin hydrogel membrane changes when lipase is present on the membrane surface.

An X-ray diffraction (XRD) analysis was performed to confirm the amorphous structure of pure pectin and the pectin hydrogel membrane. Here, the peaks for pectin were at 2Ɵ ≈ 39 and 43, with the intensities of 68 and 256, respectively. Meanwhile, the peaks obtained for pectin hydrogel membrane were at 2Ɵ ≈ 39 and 43, and the intensity decreased to 45 and 153, respectively. This can be ascribed to the more amorphous characteristic of the pectin hydrogel membrane compared to pure pectin. The diffractogram is displayed in Figure 2c. The elastic properties of the pectin hydrogel membrane were based on the result of a visual inspection, and are suitable as optical sensor matrices.

### 3.2. The Response of a Triglyceride Optical Sensor Using a Pectin Hydrogel Membrane

The construction of the TG optical biosensor consisted of the preparation of a pH optical sensor [26,32] that was modified with the addition of a lipase enzyme. Lipase will catalyze the transformation of a TG into a fatty acid, which will be detected by the pH optical sensor.

**Figure 3:**
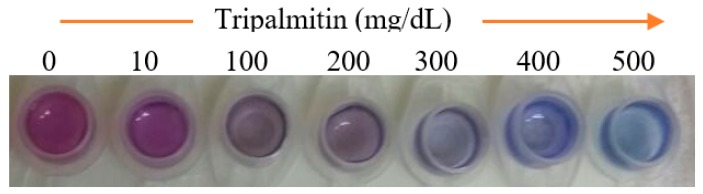
Color change of the TG biosensor at various concentration of tripalmitin (TP), a triglyceride, on a pectin hydrogel membrane.

The response of the pH optical sensor at pH 5–9 has a sensitivity of 0.056 (*R*^2^ = 0.0986), where the color change of CI takes place against the change of pH, as reported by Hasanah et al. [33]. This becomes the basis for TG determination of this optical biosensor, as a pH change will occur when lipase hydrolyzes TPs into fatty acids. The CI will undergo a change in color proportional to the increasing TP concentration as a result of this pH change, as shown by Figure 3.

### 3.3. The Effect of Buffer Concentration, pH, and Loading Enzyme Concentration against the Response of a Triglyceride Optical Biosensor 

As mentioned earlier, the upper layer of this TG biosensor consisted of a lipase enzyme solution. TP, a triglyceride, was used as the substrate for this biosensor’s characterization. A lipase enzyme will catalyze TG hydrolysis into fatty acids and glycerol [28]. The released acid results in a decrease in pH, leading to a color change on the pectin hydrogel membrane from pink to blue. The absorbance of this color change can be measured at *λ_max_* = 615 nm.

In addition, the pH change also depends on the strength of the ionic buffer [19,20]. The effect of buffer concentration against biosensor response is observed with PBS concentrations from 10–100 mM. The TG biosensor response against various PBS concentrations at pH 7 is shown in Table 1. A phosphate buffer with a concentration of 50 mM gives the best linearity and sensitivity compared to other concentrations. In this case, buffer concentration of 50 mM is the most suitable condition for lipase activation. Therefore, a phosphate buffer of 50 mM was selected as the buffer concentration for the next optimization.

The effect of environmental pH against the TG optical biosensor response at TP concentrations of 100–500 mg/dL has been evaluated, in order to determine the optimum pH of biosensor’s performance. Previous studies have reported that lipase works best at an optimal pH of between 6 and 8 [6,34].

Table 2 shows an increase of the biosensor response at pH 6–7, but a decrease at pH 7.5 and 8. The decrease in biosensor sensitivity is caused by the change in the conformation of the enzyme binding sites, thus decreasing their function in degrading triglycerides [33]. In this study, the best sensitivity was given by pH 7—hence, pH 7 is used for the upcoming characterization of the TG biosensor. This optimum pH is similar to studies reported before, which used amperometry and electrochemical methods [35,36]. However, a different optimum pH is reported by Narang et al. [37]. This difference can be caused by a different membrane matrix, type of substrate, enzyme, and immobilization method [38].

**Figure 4:**
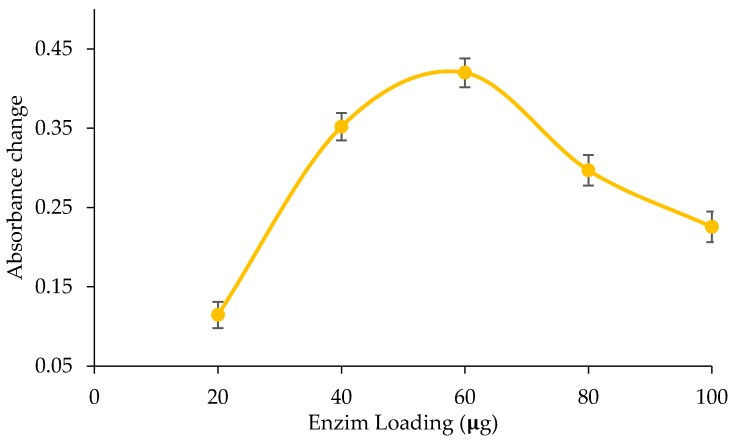
Profile lipase loading on the response biosensor.

The biosensor response also depends on the number of immobilized enzymes within the matrix. The increase of enzyme has shown to increase the biosensor response. The profile of enzyme loading is shown in Figure 4. The biosensor response increased with enzyme concentrations of 20 to 60 µg, but decreased in response to enzyme loading above 60 µg. The increase causes the rate of TP hydrolysis into fatty acids to intensify as well. On the other hand, the decrease of biosensor response is due to the extreme pH change caused by a reaction, which inactivates the enzyme binding sites [39]. The dwindling response can also be ascribed to the excessive enzyme that inhibits substrate diffusion [38,40]. The highest response was obtained at enzyme lipase loading of 60 µg.

### 3.4. Characterization of Triglyceride Optical Biosensor

**Figure 5:**
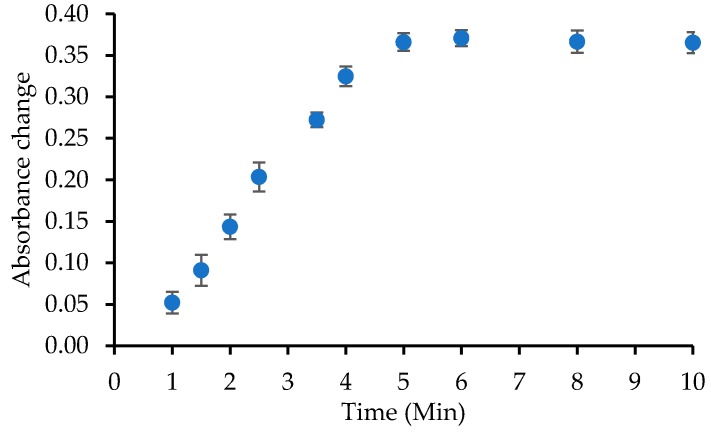
Response time of the TG biosensor.

The response time of a biosensor indicates how fast it reacts to the change of external environment. The response time of this triglycerides biosensor is measured at a TP concentration of 400 mg/dL in PBS 50 mM solution (pH 7). The determination of the response time was conducted every minute, as shown in Figure 5. The maximum time response is exhibited in the fifth minute, where afterward, the biosensor does not show any response changes. Therefore, it can be concluded that the optimal response time of this triglyceride biosensor is 5 min. The quicker the response time, the more effective the biosensor. Response time can be affected by several factors, such as diffusion, enzymatic activities, loading enzyme, and enzyme immobilization mechanism [41].

**Figure 6:**
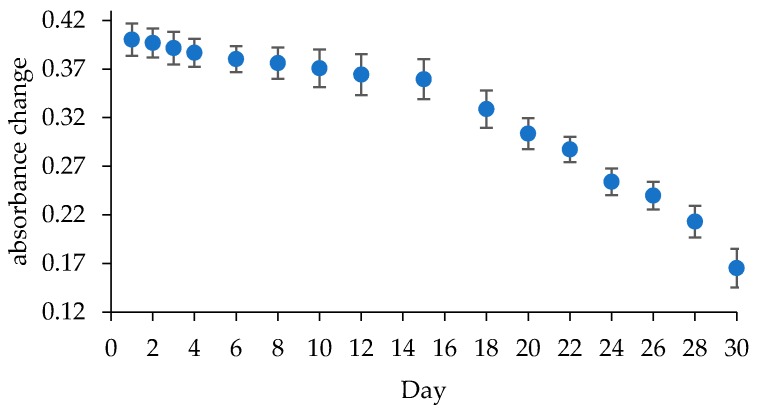
Profile of lifespan of the TG biosensor.

The determination of the biosensor lifespan is necessary to identify its stability and durability during usage. The biosensor is made with the same concentration, where 400 mg/dL of TP was added and dissolved in 50 mM PBS at pH 7. The lifespan was evaluated for 30 days. The results show that biosensor absorbance decreased 7.4% after 10 days of storage, 10.2% after 15 days of storage, and 24.2% after 25 days of storage. Therefore, it can be concluded that this TG biosensor can be used for up to 15 days of storage. The results of this lifespan study are shown in Figure 6.

### 3.5. Determination of Linearity, the Limit of Detection, and Reproducibility

**Figure 7:**
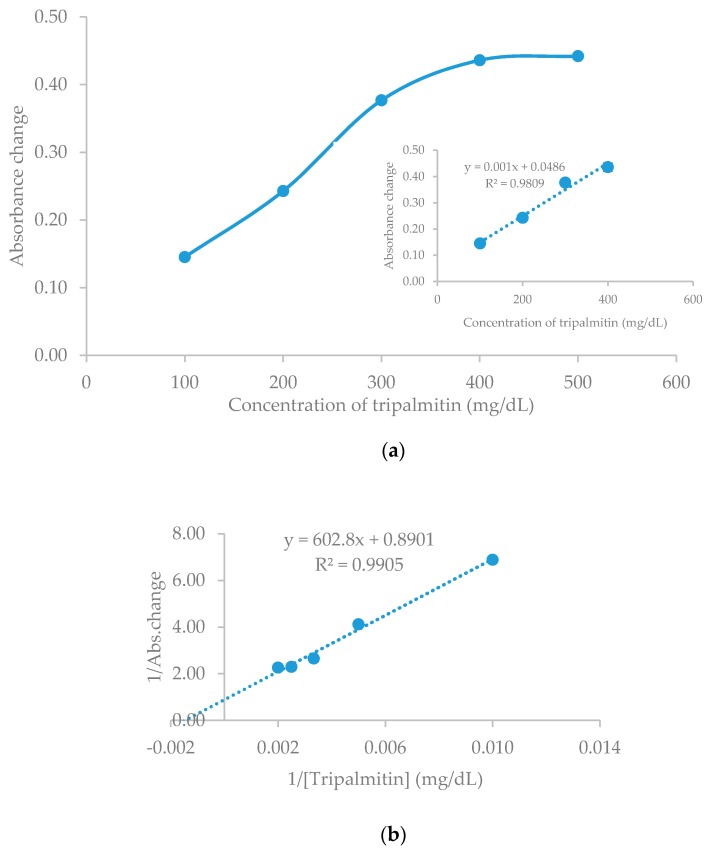
TG biosensor response against TP concentration: (**a**) Calibration curve of the TG biosensor; (**b**) Lineweaver–Burk plot of pectin hydrogel membrane-based TP concentration that is immobilized by enzyme and CI.

After all parameters that build this biosensor had been characterized, the linearity and limit of detection of this biosensor were finally determined. The biosensor response after the addition of TP from 100 mg/dL to 500 mg/dL can be observed in Figure 7a. The response of biosensor against TP increases linearly with an increasing concentration of TP. The increase of substrate in the biosensor membrane surface triggers the increase of TP degradation into fatty acids, thus causing color change on the hydrogel membrane from pink to blue. The response of the optical biosensor reaches a constant rate of 500 mg/dL, because the occupied binding sites of the enzyme have reached saturation.

An approximate linear response of TG biosensor was determined at the range of 100–500 mg/dL of TP concentration, with 0.001 sensitivity (100–400 mg/dL) and *R*^2^ = 0.981 (*n* = 4). The limit of detection (LOD) is defined as the lowest TP concentration where there is no change in optical biosensor response. The LOD of the TP is estimated to be 15 mg/dL.

The kinetic parameter of the enzymatic reaction can be calculated through a Lineweaver–Burk plot, based on the experimental data. The Michaelis–Menten (*Km*) constant calculated from Lineweaver–Burk plot was 677 mg/dL (7.6 mM), and the *Vmax* = 1.12 (Figure 7b). Some researchers have reported that the *Km* value varies with the type of substrate and lipase immobilization matrix. Minakshi and Pundir [42] reported that the *Km* value for lipase immobilized in the aryl amine matrix for the determination of a TG, using a plastic strips, was 11.6 mM. Furthermore, Narang et al. [29] determined the *Km* value of lipase in the polyvinyl chloride (PVC) matrix for the amperometric TG biosensor to be valued at 5.0 mM.

The reproducibility of a TG biosensor is measured by using 20 biosensors made with the same concentration and measured at the same time. The results indicate that this biosensor has a good reproducibility, as shown by the RSD value, which reached 2.5%. This biosensor also exhibits good linearity, with more than *R*^2^ = 0.9.

## 4. Conclusions

A TG optical biosensor based on a pectin hydrogel membrane and CI was successfully established. All parameters required for the construction of this parameter have been optimally determined, resulting in an excellent linear response range towards TP in the TG concentration range of human blood serum. This biosensor is competent in detecting TGs promptly, and displays outstanding precision. This TG biosensor will be developed further to be used on real blood serum. We strongly believe the concept that this biosensor carries can also be used to design other biosensors that can detect more health indicators.

## Figures and Tables

**Table 1:** The effect of buffer concentration against sensitivity and determination coefficient (*R*^2^) of the TG biosensor at 615 nm.

Buffer Concentration (mM)	TP Concentration (mg/dL)	Sensitivity (∆A/mg/dL)	Determination Coefficient (*R*^2^) (*n* = 4)
10	100–400	0.0007	0.9612
50	100–400	0.001	0.9807
100	100–400	0.0008	0.9585

**Table 2:** The effect of pH buffer against the sensitivity and determination coefficient (*R*^2^) of the TG biosensor at 615 nm.

pH Buffer	TP Concentration (mg/dL)	Sensitivity (∆A/mg/dL)	Determination Coefficient (*R*^2^) (*n* = 4)
6	100–400	0.0006	0.9579
6.5	100–400	0.0008	0.9665
7	100–400	0.001	0.9809
7.5	100–400	0.0007	0.9513
8	100–400	0.0005	0.9165
